# An Analysis of the Social Impacts of a Health System Strengthening Program Based on Purchasing Health Services

**DOI:** 10.1007/s44197-023-00147-8

**Published:** 2023-10-07

**Authors:** Eric Tchouaket, Hermes Karemere, Drissa Sia, Woolf Kapiteni

**Affiliations:** 1 Department of Nursing, Université du Québec en Outaouais, 5 Rue Saint-Joseph, J-2204, Saint-Jérôme, QC J7Z 0B7 Canada; 2 Regional School of Public Health, Catholic University of Bukavu, Bukavu, Democratic Republic of the Congo; 3 University of Lubumbashi and Kirotshe Higher Institute of Medical Technique, Lubumbashi, Democratic Republic of the Congo

**Keywords:** Democratic Republic of Congo, Healthcare equity, Nutrition, Strategic based financing, Impact assessment, Health system strengthening

## Abstract

Access to universal health coverage is a fundamental right that ensures that even the most disadvantaged receive health services without financial hardship. The Democratic Republic of Congo is among the poorest countries in the world, yet healthcare is primarily made by direct payment which renders care inaccessible for most Congolese. Between 2017 and 2021 a purchasing of health services initiative (Le Programme de Renforcement de l’Offre et Développement de l’accès aux Soins de Santé or PRO DS), was implemented in Kongo Central and Ituri with the assistance of the non-governmental organization Memisa Belgium. The program provided funding for health system strengthening that included health service delivery, workforce development, improved infrastructure, access to medicines and support for leadership and governance. This study assessed the social and health impacts of the PRO DS Memisa program using a health impact assessment focus. A documentary review was performed to ascertain relevant indicators of program effect. Supervision and management of health zones and health centers, use of health and nutritional services, the population’s nutritional health, immunization levels, reproductive and maternal health, and newborn and child health were measured using a controlled longitudinal model. Positive results were found in almost all indicators across both provinces, with a mean proportion of positive effect of 60.8% for Kongo Central, and 70.8% in Ituri. Barriers to the program’s success included the arrival of COVID-19, internal displacement of the population and resistance to change from the community. The measurable positive impacts from the PRO DS Memisa program reveal that an adequately funded multi-faceted health system strengthening program can improve access to healthcare in a low-income country such as the Democratic Republic of Congo.

## Introduction

Access to healthcare is a fundamental right for all populations, including the most disadvantaged. According to data from the Democratic Republic of Congo’s (DRC) 2014 Demographic and Health Survey (DHS), more than 50% of the population had an economic well-being index in the lowest quintiles [1, 2]. According to the same data, 31% of women and 14% of men aged 15–49 were unemployed in 2013. Congolese were largely self-employed in the agricultural sector (60% of men and 65% of women), followed by sales and services, which represented 18% of men and 28% of women. The organized sector employed 5% in public administration, 1.4% in the partly state-controlled sector, and 1.2% in the formal private sector. In 2018, employment was dominated by the informal sector which represented somewhere between 81% and 98% of the country’s economy [3]. Congolese are among the poorest people in the world, and in 2021 the country ranked 179 out of 185 on the United Nations Development Programme`s Human Development Index [4]. The index represents the possibility of achieving knowledge, a decent standard of living and a long and healthy life. In 2021, the Anker Institute calculated the DRC living income (which a ‘typical family in rural DRC needs to cover the monthly cost of a basic but decent standard of living’) at US $170 or 380,000 CDF [5]. However, estimates of 2021 household monthly income in the DRC vary far below this number, from 54,224 CDF (US $24) to 338,567 CDF (US $150) [5].

Although some progress has been made in recent years, the health situation in the DRC is dire. Indeed, according to figures from the 2014 DRC’s statistical yearbook, the infant and child mortality rate was 65 per thousand, the infant mortality rate 41 per thousand, only 70.6% of children aged 12–23 months had full immunization coverage, and nearly 59% of children under 5 years of age were stunted due to malnutrition. These findings confirm the precarious economic and health status of the population. Moreover, according to the 2014 RDC’s DHS survey, more than 97% of men and 93% of women aged 15–49 had no medical insurance. Over 60% of the population did not access health services due to lack of finances, and 28.8% due to the inability to travel to receive care. This level of poverty reflects an existing financial inaccessibility which is undoubtedly the main problem in accessing healthcare services. Based on 2020 World Bank data for the DRC, health expenditures represented 4.1% of the gross domestic product (GDP). Of total current health expenditures, domestic government spending accounted for 16.1%, external funding 37.5%, out-of-pocket payments represented 39.7%, and private health insurance 6.7%.

To address the issue of affordability, country governments have considered appropriate health financing systems that can improve access to healthcare. Several methods currently being developed in the DRC include subsidized flat-rate pricing; purchasing health services approach; performance-based financing; community-based health insurance and direct payment. Direct payment for healthcare is still the most predominant form of financing as it makes care in healthcare facilities marketable. However, this is contrary to the principles of universal health coverage (UHC) as advocated by the WHO [6] as it leads to the exclusion of service users who are poor or destitute, which in this case represent 70–80% of the population who work in the informal sector [2]. The concept of UHC is multi-dimensional and encompasses concepts of population coverage, universal access to quality care and importantly financial protection against the risk of spending out-of-pocket for healthcare. Universality only happens, however, when the coverage is accessible and acceptable. Real access to services is ensured when installations and healthcare professionals are within a reasonable geographic distance and have systems in place to organize and see clients. Financial implications of access include indirect and opportunity costs related to searching out care. Finally, acceptable care is defined in terms of appropriate client- provider interactions that consider the cultural needs of the population.

One attempt to provide better healthcare coverage in the DRC was The Program for Strengthening the Supply and Developing Access to Health Care (Le Programme de Renforcement de l’Offre et Développement de l’accès aux Soins de Santé or PRO DS), financed by the European Union and implemented between 2017 and 2021 by the NGO Memisa Belgium (PRO DS Memisa) in the provinces of Kongo Central and Ituri in the DRC. Memisa is a non-governmental medical organization based in Belgium that supports access to universal health coverage for vulnerable populations worldwide. Their aim is to improve the quality of healthcare with a focus on maternal and child health. Memisa is most active in African countries, and especially in the DRC, a former Belgian colony. Memisa Belgium’s initiatives in the DRC include (a) providing infrastructure for healthcare in the form of newly built or renovated health clinics; (b) providing health supplies including medicine, equipment and blood banks; (c) offering training and support for those with malnutrition; (d) employing over 50 local professionals; and (e) providing ongoing medical training for healthcare professionals. Memisa Belgium advocates for Congolese to be aware of their own right to healthcare and works in collaboration with the Ministry of Health to recognize the needs of the population. Finally, Memisa also provides financial support by ensuring there are systems in place (copayments or solidarity funds) to ensure access to care.

Memisa’s objective in the DRC was to facilitate access to UHC, particularly for the most vulnerable, to participate in the fight against poverty and encourage health equity by providing a health service purchasing approach to healthcare and the management of malnutrition. Purchasing healthcare services is a strategic purchasing policy that includes a bundle of services that can vary widely, consisting of a series of elements that importantly include: "purchased" results (quantitative indicators and quality measures); associated performance bonuses; mechanisms for verification and cross-checking of results; governance and accountability structure; and any related support (e.g., training, information system strengthening). The success of this approach is strongly based on the involvement of the various stakeholders that include governments, suppliers and patients [7] and sufficient remuneration of human resources [8, 9]. The implementation of PRO DS Memisa offered promise for UHC implementation, it aimed to ensure real social protection and improve access to healthcare. It was therefore essential to assess the social impact of this program.

### Study Aim

This study aims to assess the social impacts of, and changes brought about by, the PRO DS Memisa program, a strategic purchasing policy to strengthen healthcare systems. It evaluates the program in the provinces of Kongo Central and Ituri.

### Pro DS Memisa

#### Objectives Targeted by the Program

**Fig. 1:**
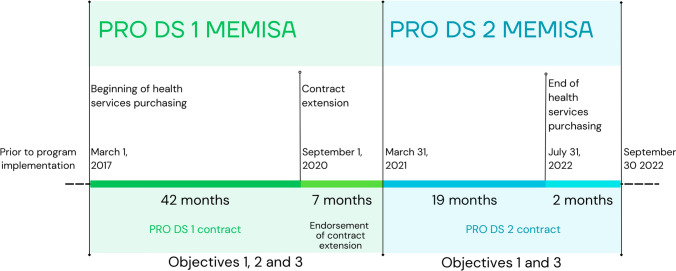
Timeline and Objectives of the PRO DS Memisa program from March 1, 2017 to September 30, 2022

**Fig. 2:**
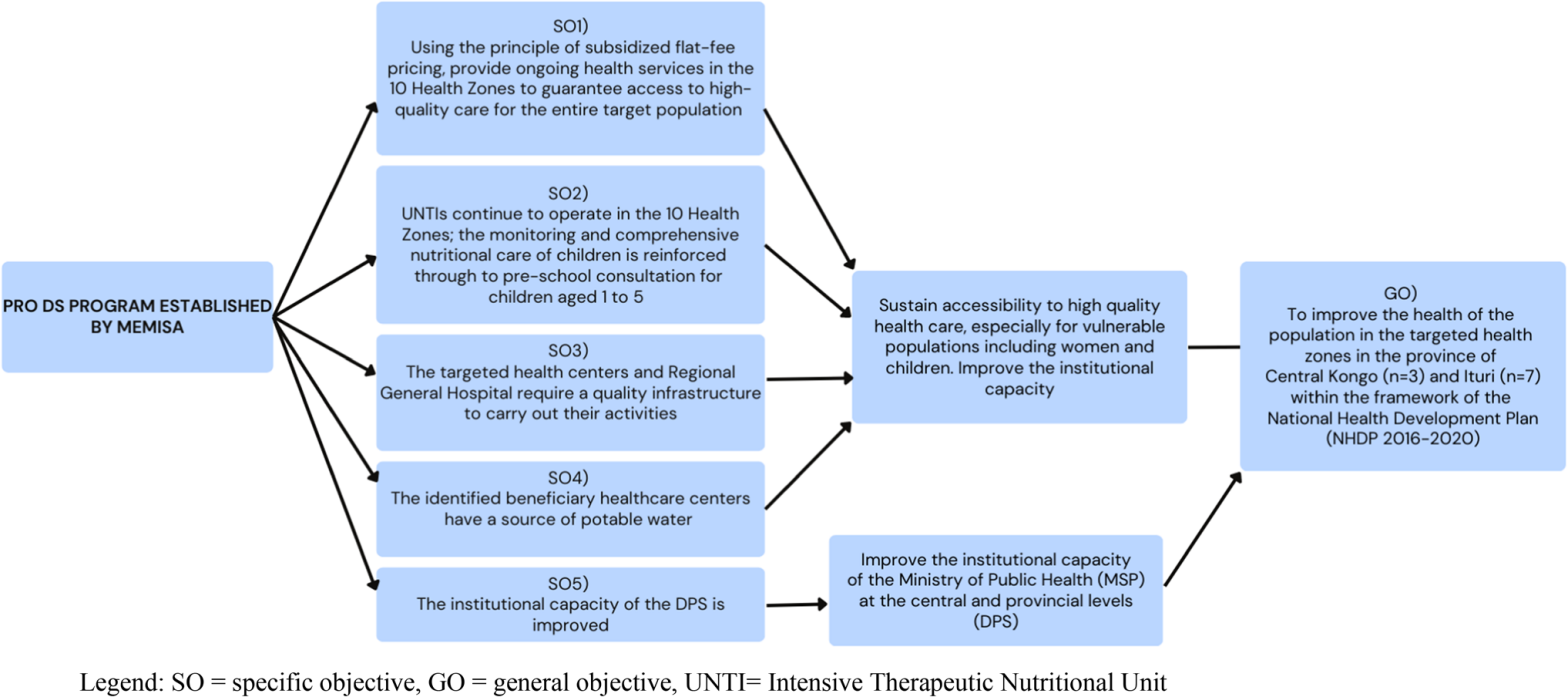
Relationship chains of the PRO DS Memisa program

The initial contract for PRO DS Memisa (PRO DS Memisa 1) implementation was signed on March 1, 2017, between the European Union's Cooperative Food Empowerment Directive (COFED) and the NGO Memisa for a period of 42 months. It had three objectives: (1) to improve the population’s quality of health in targeted health zones (HZ) and to ensure its sustainability; (2) to integrate humanitarian interventions in the health zones and (3) to improve the institutional capacity of the Ministry of Public Health at the central level and provincial level (Division provinciale de la santé or DPS). The initial contract was extended for 7 months until March 31, 2021, to integrate the quality assessment of health zones, health centers (CS) and a nutrition component in both provinces. In addition, due to COVID-19 and the urgent health measures implemented by other partners, a second PRO DS Memisa 2 contract was signed for a 19-month period spanning April 1, 2021 to September 30, 2022. This phase of the program focused on the intensification of activities to achieve objectives 1 and 3 described above. Figure 1 illustrates the timeline of the program while Fig. 2 shows the relationship between objectives.

*Objective 1)* To improve the population’s quality of health in the targeted HZ and to ensure its sustainability.

*Objective 2)* To integrate the HZ supported by humanitarian and vertical interventions.

*Objective 3)* To improve the institutional capacity of the Ministry of Public Health at the central level and provincial level.

#### Key Stakeholders and Direct Beneficiaries of the PRO DS Memisa Program

PRO DS Memisa was implemented in three of 31 HZ in Kong Central province and seven of the 36 HZ in Ituri province. The HZ chosen represented areas with very poor healthcare services and were identified under the 11th European Union funding. The main actors in each of the two provinces were: the DPS, the general referral hospitals (HGR) in each HZ (three in Kongo Central and seven in Ituri), and all CS responsible for a geographic region in the zones concerned. Contracts were signed between the regional offices and the DPS, and with the public utility institutions of the health services procurement fund (Utilité publique de fonds d’achat de services de santé or EUP-FASS). In Kongo Central, the EUP-FASS was supported by MUSAKIS (Mutuelle de santé de Kisantu). Together with the DPS, an advising physician, the EU, and technical assistants, MUSAKIS acted as an EUP-FASS under the name MUSAKIS-FASS within the framework of this project. The role of the MUSAKIS-FASS in Kongo Central and the EUP-FASS in Ituri was to contract with the HZ and CS to verify and pay their invoices. To fulfill the nutritional objectives of PRO DS Memisa 1 and PRO DS Memisa 2, contracts were signed between the regional offices and organizations for the preparation and distribution of local food. Finally, under calls for open contracts, relevant organizations were contracted to build, rehabilitate, and equip general hospitals and health centers.

In both Ituri and Kongo Central, a Memisa operating grant made it possible for the DPS to strengthen its ability to supervise the 10 targeted HZ and plan and oversee initiatives being put into place. DPS staff received a salary bonus and specific emergency activities were carried out. The program supported the DPS in improving the quality of care and services offered in these zones through: (1) quality assessment, management (supporting the development of management plans, operational action plans and drug management); (2) training, upgrading skills and providing internships; (3) facilitating the development of community initiatives and recruiting community outreach workers.

The MUSAKIS FASS in Kongo Central and EUP-FASS in Ituri entered into contracts with the HZ, HGR and CS for the purchase of services between September 1st, 2017, and July 31st, 2022, to carry out the program’s activities.

For the HZ, the HGR and health zone management team (ECZ), these contracts stipulated that the program provide: (1) supervision and guidance at the management level for the ECZ and management supervision for the HZ; (2) management of the EZS and top-up/supplementary remuneration; (3) purchase of services for the HGR in the form of third-party payments; (4) a bonus for the HGR depending on the quality of care and; (5) a co-payment to supplement the total cost of care for the most disadvantaged patients in the HGR (maximum 5% of the budget). The budget for the ECZ (points 1 and 2 above) was allocated as follows: 50% for supplementary remuneration; 35% for the purchase of medication, laboratory reagents and medical consumables paid directly to the relevant medicine distribution centers in the program's HZ; 10% for ECZ operating costs; and 5% for reserves/savings.

The CS were initially responsible for: (1) the purchase of services at the agreed fixed cost for prenatal and preschool consultations and vaccinations for pregnant women and children aged 0–5 years; (2) the purchase of services via third-party payment at the agreed fixed cost for childbirth and delivery; (3) the purchase of services via third-party payment at a flat-rate cost for childbirth, delivery and postnatal care; (4) co-payment coverage for indigent women (maximum 5%); (5) co-payment coverage for indigent pregnant women (maximum 3%); and (6) offering a quarterly bonus according to the level of quality of care provided. Health centers receive a sum of $0.5 per health area resident based on the area’s 2019 population.

The direct beneficiaries of PRO DS Memisa were the HZ; the two DPS of Kongo Central and Ituri; the EUP-FASS of Ituri and the MUSAKIS-FASS in Kongo Central; the CAAMEKI (Kisantu—Kongo Central) and the CADIMEBU (Ituri); the population of the three HZs (*n* = 486,020) (Gombe-Matadi, Kisantu and Ngidinga) in Kongo Central and the population of the seven HZs (*n* = 1,142,351) (Bunia, Nyankunde, Komanda, Mambasa, Tchomia, Nizi and Drodro) in Ituri.

The indirect beneficiaries of the program were the entire population of the two provinces, a total of approximately 10,981,289 inhabitants (4,373,898 inhabitants in Kongo Central and 6,607,391 inhabitants in Ituri). All population statistics are from 2020.

### Theoretical Framework

#### Health Impact Assessments

Health impact assessments are defined as: “a combination of procedures, methods and tools that systematically judges the potential, and sometimes unintended, effects of a policy, plan, program or project on the health of a population and the distribution of those effects within the population” [10]. By monitoring and measuring the impact of a program, valuable data can be provided to decision-makers on how to best maximize the resulting positive impacts and minimize any negative impacts appropriately.

To meet the objective of the study, we focused on the health and social outcomes brought about by PRO DS Memisa, measuring levels of supervision provided to health centers, changes in health and nutritional status of the population and involvement and uptake of the program in the community. To measure effect, this study used a controlled longitudinal model to compare between areas that were served by PRO DS Memisa and those that were not.

#### Research Questions

At each stage of the study the following research questions were analyzed:

In step 1, to define the scope of the analysis:

(i) Who are the stakeholders?
(ii) Who was affected by the project (beneficiaries)?
(iii) Who had an impact on the project?
(iv) What do we expect to change for the intended beneficiaries?

In step 2, to produce a map of outcomes based on stakeholder meetings:

(i) What are the project’s resources or inputs?
(ii) What are the activities carried out within the framework of this project?
(iii) What are the results or products of this project?

In step 3, we define the results indicator:

(i) How does one measure the changes observed?
(ii) What is the quantified result of this observed change?

In step 4, we consider the possible impact of the PRO DS Memisa program:

(i) What would have happened without the action of the project?
(ii) How much of the change is outside of the project’s effects?
(iii) Can the result diminish or increase over time?

## Methodology

### Design and Data Sources

This study is a retrospective study incorporating a documentary review and a secondary analysis of longitudinal quantitative data comparing the 10 health zones supported and the 49 health zones not supported by the PRO DS Memisa program.

### Documentary Review

At the start of the study an analysis grid was developed to guide the documentary review. This grid’s content has been validated by researchers and field teams and made it possible to collect information on the available resources, the activities carried out, and the results that were expected and achieved. Documents underwent content analysis based on the theoretical framework of health impact assessments that made it possible to construct the chain of potential effects of the program. The research team and the implementation team identified relevant existing documents to analyze; all documents were provided by Memisa. A total of 48 documents were consulted for Kongo Central and 31 for Ituri. Some documents were common to the two provinces, in particular project documents such as annual reports and signed contracts. The complete list of documents is available in supplementary file1.

### Quantitative Secondary Data from PRO DS Health Zones and Non-PRO DS Health Zones

**Fig. 3:**
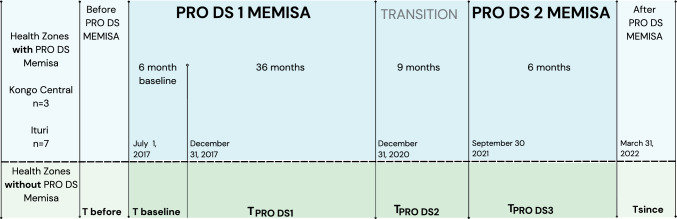
Time periods used in the comparative longitudinal approach to analyzing changes due to the PRO DS Memisa program

The secondary data were obtained from the annual reports of the provincial health division (Division provinciale de la santé or DPS), the PRO DS Memisa reports, and the national health information systems (systèmes nationaux d’information sanitaire or SNIS) from all HZ in both provinces. Figure 3 shows the diagram of the comparative longitudinal approach used to analyze changes that resulted from the PRO DS Memisa program. Data were collected in five stages: (1) before implementation of the program in 2016 (*T*_before_); (2) in the first year of the program (baseline in 2017) (*T*_baseline_); (3) during the first 36 months of PRO DS Memisa 1 (1st January, 2018 to 31st December, 2020) (*T*_PRO DS 1_); (4) during the final 9 months of PRO DS Memisa 1 while in transition to PRO DS Memisa 2 (1st January, 2021 to 30th September, 2021) (*T*_PRO DS 2_); (5) during the most recent 6 months of PRO DS Memisa 2 (1st October 2021, to 31st March, 2022) (*T*_PRO DS 3_).

### Operational Definitions of Indicators of Change Due to PRO DS Memisa

In line with PRO DS Memisa objectives, 74 indicators for Central Kongo and 65 for Ituri were identified and collapsed into six impact domains: (1) Supervision and management of health zones and health centers; (2) Use of health and nutritional services; (3) Nutritional health; (4) Immunization; (5) Reproductive and maternal health; and (6) Newborn and child health (see Table 1). These indicators were those set out by the SNIS that measured maternal and child key health outcomes. The data were extracted for both provinces from the SNIS when available for the period of 2017–2021. If an indicator had missing data for more than 2 years, it was excluded from the analyses.

**Table 1:** List of indicators used for the analysis of social impacts or changes due to the PRO DS Memisa program

Areas	Modified indicators	Ituri	Kongo Central
Supervision and management of health zones and healthcare centers Kongo Central: 4 Ituri: 3	Proportion of health center supervision	X	X
	Proportion of CODESA meetings held, with minutes taken	X	X
	Proportion of weekly meetings held by health zone management team	X	X
	CODESA rate of decision making in previous month	X	X
Use of healthcare and nutritional services Kongo Central: 4 Ituri: 3	Admissions at start of month (carry over from previous month) UNTA-Female	X	X
	Admissions at start of month (carry over from previous month) UNTI-Female	X	X
	Further referral to an UNTA	X	X
	Acute moderate malnutrition at the supplemental nutritional unit	X	X
	Acute moderate malnutrition transferred to UNTA/UNTI	X	X
	Referred to UNTI	X	X
	Referred to UNTA	X	X
	Transferred to another UNTA		X
	Postoperative infection(s)	X	X
	Cases referred to hospital	X	X
	Average hospital stay (days)	X	X
	Bed occupancy rate	X	X
	Curative service (primary care, treatments) utilization rate	X	X
	Attendance at preschool medical consultation	X	
	Hospital mortality rate (48 h post admission)		X
Nutritional health Kongo Central: 9 Ituri: 8	Attrition (failure to follow up) at the Supplemental Nutritional Unit	X	X
	Attrition (failure to follow up) at UNTI	X	X
	Other admission with complications-Female		X
	Deaths at the Supplemental Nutritional Unit	X	X
	Deaths at UNTA	X	X
	Deaths at UNTI	X	X
	Discharged (healed from malnutrition)	X	X
	Healed from malnutrition—from an UNTA	X	X
	Healed from malnutrition—from an UNTI	X	X
Vaccination Kongo Central: 7 Ituri: 7	Penta 1-Penta 3 attrition (failure to present for booster)	X	X
	Vaccinated for PCV-13	X	X
	Vaccinated for VAA	X	X
	Vaccinated for BCG	X	X
	Vaccinated for Pentavalent 1	X	X
	Vaccinated for Pentavalent 3	X	X
	Vaccinated for VAR	X	X
Reproductive and maternal health Kongo Central: 19 Ituri: 19	Combined oral contraception pill pack	X	X
	Progesterone implant	X	X
	Progesterone only pill pack	X	X
	Pregnant women who consulted prenatal care services	X	X
	Pregnancy related edema with complications	X	X
	Pregnancy related edema	X	X
	*Z* score of − 3 (weight for height) or arm circumference less than 115 mm with complications	X	X
	*Z* score of − 3 (weight for height) or arm circumference less than 115 mm		X
	Postpartum relapse with complications	X	X
	Postpartum relapse		X
	Proportion of pregnant women who received at least two anti-malarial treatments (sulfadoxine/pyrimethamine oral)	X	
	Proportion of pregnant women who received a long-lasting insecticidal net during their prenatal care visit #1	X	X
	Proportion of second post-natal consultations	X	X
	Proportion of pregnant women who received the 3rd dose of folic acid	X	X
	Assisted delivery rate	X	X
	Prenatal 1 consultation rate	X	X
	Prenatal consultation rate at week 16	X	X
	Prenatal 4 consultation rate	X	X
	Tetanus vaccine coverage in pregnant women	X	X
	Hospital mortality rate (48 h since admission)	X	
	Rate of applicants using modern contraceptive methods	X	X
Newborn and child health Kongo Central: 21 Ituri: 14	Live births	X	X
	Proportion of children who received long-lasting insecticidal net during prenatal consultations	X	X
	Proportion of children < 5 years old with acute malnutrition treated as per the national protocol: PCIMA	X	X
	Proportion of children < 5 years old with diarrhea treated with oral rehydration solution, zinc	X	X
	Proportion of children < 5 years old with malaria treated according to the national protocol	X	X
	Proportion of children < 5 years old with pneumonia who received antibiotics	X	X
	Proportion of children aged 6 to 59 months with moderate acute malnutrition	X	X
	Proportion of children aged 6 to 59 months with severe acute malnutrition	X	X
	Proportion of children aged 6–23 months still being breastfed	X	X
	Proportion of children treated for simple diarrhea		X
	Proportion of children treated for simple pneumonia		X
	Proportion of newborns put to the breast within 1 h of birth	X	X
	Proportion of newborns who received 5 components of Essential Newborn Care and three examinations in the first 6 days of life	X	X
	Proportion of children under 6 months exclusively breastfed	X	X
	Proportion of live newborns with low birth weight	X	X
	Proportion of live newborns with very-low birthweight (less than 2500 g)	X	X
	Infant and child hospital incidence of diarrhea		X
	Infanto-juvenile hospital incidence of pneumonia		X
	Attendance at preschool consultation		X
	Case fatality rate for severe pneumonia in children under the age of five		X
	Infant and child fatality rate from diarrhea with severe dehydration		X
Grand total		**65**	**74**

### Data Analysis

Documents were analyzed to identify the program elements implemented that strengthened the supply and promoted the access to healthcare services. The results provided a better understanding of the logic and stages of program implementation in the two provinces.

**Fig. 4:**
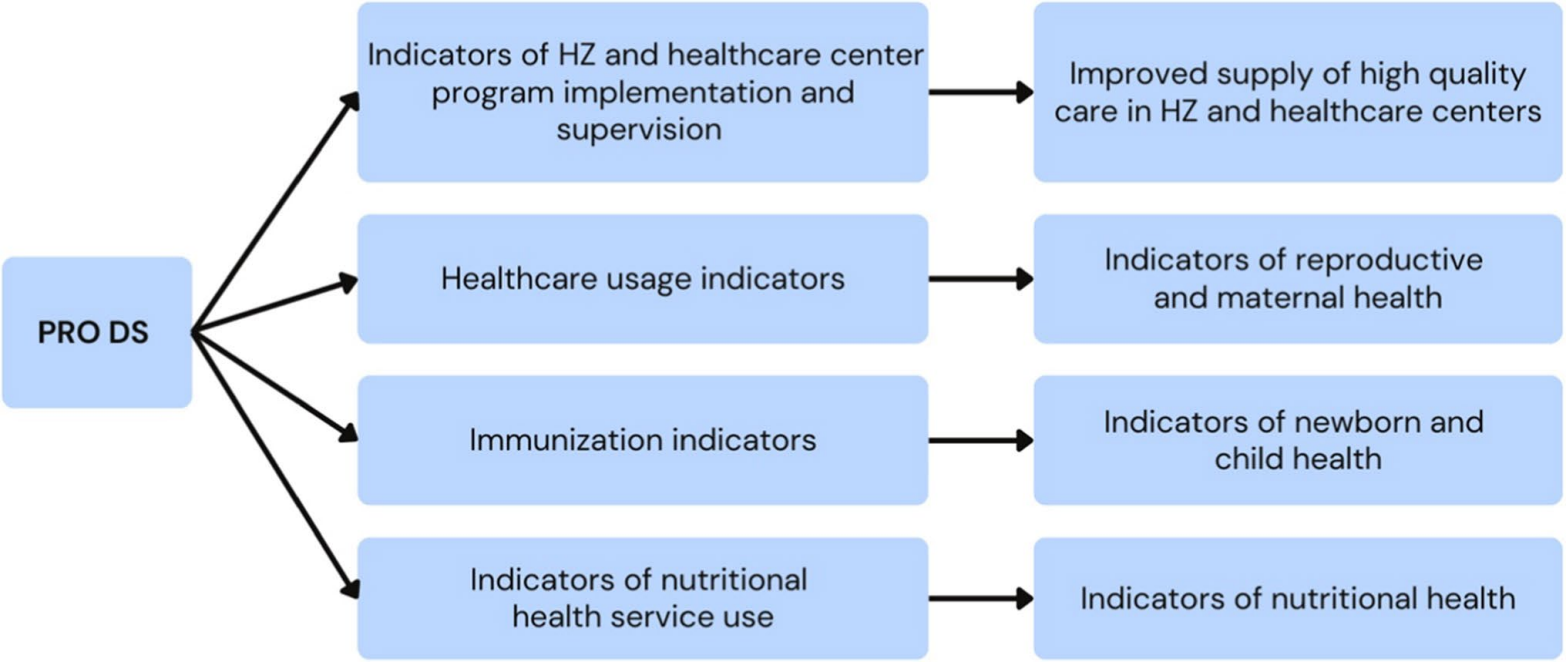
Causal chain of relationships between indicators of impact of the PRO DS Memisa program

Quantitative analyses of the effects of the program were carried out in two phases. As noted above, indicators were identified from the SNIS, the Memisa PRO DS activity reports, and the DPS. A causal change of relationships was interpreted from these indicators (Fig. 4). Estimation of the effects was made by comparing the results of indicators over time between the 10 HZ covered by PRO DS Memisa and those not covered by PRO DS Memisa. The impact of the program on a health indicator was measured using a difference in difference equation [11] where the level of the indicator (Ind) was compared for PRO DS(_PRO DS_) health zones and non-PRO DS _(non PRO DS)_ health zones, and for the periods before the program began, and at baseline (*T*_before_ and *T*_baseline_), denoted Ind _before_, and PRO DS times 1,2 and 3, since _(TPRO DS1, TPRO DS2, TPRO DS3)_ denoted Ind_since_. The rate of change or percentage impact (% impact) was defined as follows:

$$\begin{gathered} {\text{Impact on the indicator}}\;{\text{ = }}\;{\text{Double difference of the indicator}} \hfill \\ {\text{ = (Ind }}T_{{{\text{since}}}} - {\text{Ind }}T_{{{\text{before}}}} )_{{{\text{PRO~DS}}}} - {\text{(Ind }}T_{{{\text{since}}}} - {\text{Ind }}T_{{{\text{before}}}} )_{{{\text{Non~PRO~DS}}}} \hfill \\ \% {\text{Impact}} = \left[ {\left( {\frac{{\left( {{\text{Ind}}_{{{\text{since}}}} - {\text{Ind}}_{{{\text{before}}}} } \right)_{{{\text{PRO~DS}}}} }}{{\left( {{\text{Ind}}_{{{\text{since}}}} - {\text{Ind}}_{{{\text{before}}}} } \right)_{{{\text{Non~PRO~DS}}}} }} - 1} \right) \times 100} \right] \hfill \\ \end{gathered}$$

Analyses were performed using Microsoft Excel and SPSS-26 software.

## Results

### Principal Activities Planned Under the PRO DS Memisa Program

**Fig. 5:**
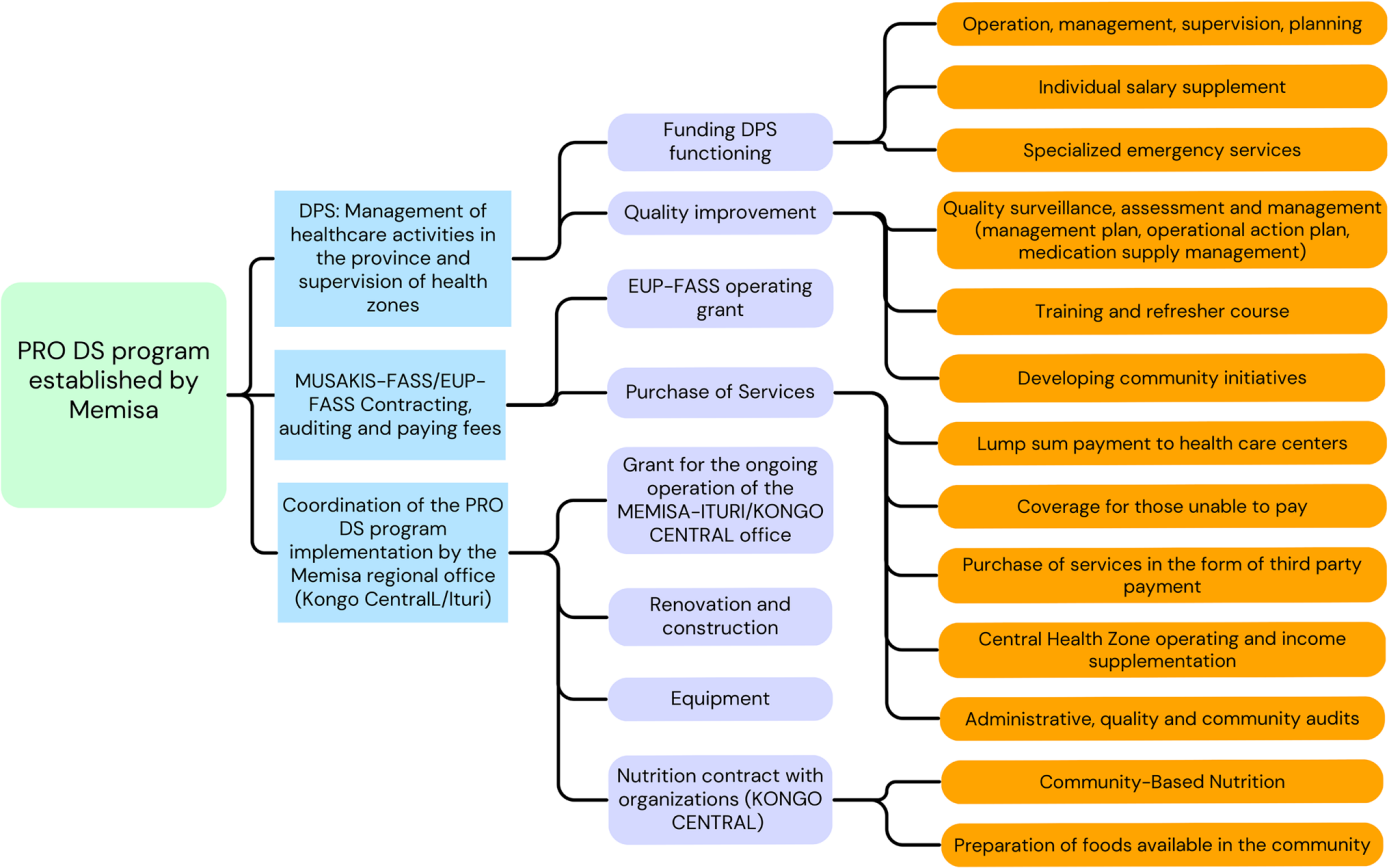
Main activities of the PRO DS Memisa program according to the stakeholders

The organizational structure and main activities of PRO DS Memisa in both provinces is shown in Fig. 5. It was informed by stakeholders across both provinces.

### Positive Effects of the Program Between March 1, 2017 and September 30, 2022

#### Positive Effects of the Program: Supervision and Management of Health Zones and Health Centers

In the province of Kongo Central, PRO DS Memisa contributed to an increase in the amount of health center supervision, health region development committee (*comité de develppement de l’aire de santé* or CODESA) meetings with recorded minutes, and weekly HZ management team meetings. These increases were 15%, 56%, and 56%, respectively in the three PRO DS Memisa HZ compared to the non-PRO DS HZ.

In Ituri, no changes were observed from program implementation in terms of supervision and management of HZ and health centers in the PRO DS HZ compared to non-PRO DS HZ.

#### Positive Effects of the Program: Health and Nutrition Services

In Kongo Central, the effects on health and nutrition services seen from program implementation in the three PRO DS Memisa HZ compared to the non-PRO DS HZ are presented in Table 2. Outcomes included:

- Cases referred to hospital increased by a factor of 3.5;
- Access to treatment increased by 56%;
- Postoperative infections decreased by 70%;
- A strong uptake was witnessed in the use of nutritional services: there was a 21.3-fold increase in transfers to a therapeutic outpatient nutrition unit (UNTA) which then grew to an 85.1-fold increase in further referrals to another UNTA. The was also a 26.9-fold increase in admissions to an UNTA with a concordant 4.3-fold increase admitted to intensive therapeutic nutrition units (UNTI).

**Table 2:** Positive impacts of the PRO DS Memisa program in Kongo Central

Areas	Indicators	PRO DS Memisa		Non PRO DS Memisa		Difference in difference estimation		
		Baseline monthly average	Project period 2018–2021 monthly average	Baseline monthly average	Project period 2018–2021 monthly average	% Impact (Difference PRO DS/Difference non PRO DS) − 1	Interpretation	
							Fold increase or decrease	Literal
Supervision and management of health zones and healthcare centers	Proportion of healthcare center supervision	6.4	9.7	6.5	9.3	0.15	0.15	0.15 times more
	Proportion of CODESA meetings held, with minutes taken	8.3	11.1	7.9	9.7	0.56	0.56	0.56 times more
	Proportion of weekly meetings held by health zone management team	5.5	8.3	6.6	8.4	0.56	0.56	0.56 times more
Use of healthcare and nutritional services	Proportion of postoperative infection(s)	0.1	0.2	0.2	0.4	− 0.70	− 0.30	0.3 times less
	Bed occupancy rate	10.1	11.1	4.4	5.2	0.25	0.25	0.25 times more
	Cases referred to hospital	0.4	0.7	0.2	0.3	3.46	3.46	3.46 times more
	Curative service (primary care, treatments) utilization rate	3.3	5.2	3.3	4.5	0.56	0.56	0.56 times more
	Average hospital stay (days)	67.2	133.5	57.4	135.1	− 0.15	− 0.85	0.85 times less
	Transferred to another UNTA	0.1	0.2	0.4	0.4	21.29	21.29	21.29 times more
	Further referral to an UNTA	2.6	6.7	0.5	0.6	85.11	85.11	85.11 times more
	UNTA Admissions at start of month (carry over from previous month) -Female	9.7	47.8	6.3	7.6	26.89	26.89	26.89 times more
	UNTI Admissions at start of month (carry over from previous month) -Female	1.9	4.4	1.5	2.0	4.31	4.31	4.31 times more
Nutritional health	Deaths at UNTA		0.2	0.2	0.2	− 0.15	− 0.85	0.85 times less
	Attrition (failure to follow up) at the Supplemental Nutrition Unit	1.0	0.8	0.3	0.5	− 1.89	− 1.89	1.89 times less
	UNTI Attrition (failure to follow up)	0.5	0.4	0.3	0.4	− 1.64	− 1.64	1.64 times less
	Discharged (healed from malnutrition)	1.8	5.6	1.3	1.5	12.66	12.66	12.66 times more
	UNTI Deaths	0.1	0.0	0.1	0.1	− 0.02	− 0.98	0.98 times less
Vaccination	Vaccinated for BCG	5.3	8.9	5.1	8.2	0.16	0.16	0.16 times more
	Vaccinated for PCV-13	4.5	8.9	5.3	8.6	0.32	0.32	0.32 times more
	Vaccinated for VAA	4.3	8.6	5.2	8.3	0.38	0.38	0.38 times more
	Vaccinated for Pentavalent 1	5.2	9.7	6.1	9.2	0.45	0.45	0.45 times more
	Vaccinated for Pentavalent 3	4.7	8.9	5.7	8.6	0.45	0.45	0.45 times more
	Vaccinated for VAR	4.5	8.6	5.5	8.3	0.45	0.45	0.45 times more
	Penta 1-Penta 3 dropout rate	0.7	0.7	0.6	0.6	− 0.35	− 0.65	0.65 times less
Reproductive and maternal health	Progesterone Only Pill Pack	0.6	0.9	1.7	1.8	1.63	1.63	1.63 times more
	Pregnant women who consulted prenatal care services	962.6	1,244.6	807.7	850.1	5.65	5.65	5.65 times more
	Proportion of pregnant women who received the 3rd dose of folic acid	7.4	9.0	7.8	9.4	0.00	0.00	No change
	Prenatal 1 consultation rate	5.1	8.9	5.9	7.5	1.46	1.46	1.46 times more
	Prenatal 4 consultation rate	2.2	4.6	2.2	3.4	1.14	1.14	1.14 times more
	Tetanus vaccine rate of coverage in pregnant women	4.6	8.3	5.7	7.5	1.04	1.04	1.04 times more
	Assisted delivery coverage rate	4.4	7.7	5.0	6.3	1.71	1.71	1.71 times more
	Pregnancy related edema with complications	0.3	0.9	0.4	0.4	− 62.52	− 62.52	62.52 times less
	Pregnancy related edema	0.9	2.7	0.7	0.6	− 30.02	− 30.02	30.02 times less
Newborn and child health	Proportion of children < 5 years old with acute malnutrition treated as per the national protocol: PCIMA	3.2	10.1	7.8	7.4	− 19.40	− 19.40	19.4 times less
	Proportion of children < 5 years old with diarrhea treated with oral rehydration solution, zinc	7.1	9.4	6.3	8.0	0.40	0.40	0.4 times more
	Proportion of children < 5 years with pneumonia who received antibiotics	7.8	9.9	7.3	9.0	0.25	0.25	0.25 times more
	Proportion of children < 5 years old with malaria treated according to the national protocol	7.7	9.7	8.1	9.7	0.14	0.14	0.14 times more
	Proportion of live newborns with low birth weight	0.7	0.6	0.8	0.6	− 0.62	− 0.38	0.38 times less
	Proportion of live newborns with very-low birthweight (less than 2500 g)	0.7	0.6	0.8	0.6	− 0.62	− 0.38	0.38 times less
	Proportion of children aged 6 to 59 months with moderate acute malnutrition	0.4	0.4	0.2	0.2	− 5.22	− 5.22	5.22 times less
	Proportion of children aged 6–23 months still being exclusively breastfed	5.6	8.2	6.0	8.2	0.19	0.19	0.19 times more
	Attendance at preschool consultation	4.4	8.8	4.4	8.6	0.03	0.03	0.03 times more
	Proportion of children treated for simple diarrhea	7.1	9.4	6.4	8.1	0.36	0.36	0.36 times more
	Proportion of children treated for simple pneumonia	7.8	9.9	7.3	9.0	0.28	0.28	0.28 times more
	Infant and child hospital incidence rate of diarrhea	0.8	0.9	0.7	0.6	− 2.33	− 2.33	2.33 times less

In Ituri, the effects on health and nutrition services seen from program implementation in the seven PRO DS versus non-PRO DS HZ are presented in Table 3. Outcomes included:

- The proportion of cases referred to hospital increased by 41%;
- Curative service use increased by a factor of 1.2;
- There was a 35% increase in attendance in preschool consultations;
- The average hospital length of stay decreased by 29%;
- Hospital bed occupancy rate decreased by 91%;

**Table 3:** Positive impacts of the PRO DS Memisa program in Ituri

Areas	Indicators	PRO DS Memisa		Non PRO DS Memisa		Difference in difference estimation		
		Baseline monthly average	Project period 2018–2021 monthly average	Baseline monthly average	Project period 2018–2021 monthly average	% Impact (Difference PRO DS/difference non PRO DS) − 1	Interpretation	
							Fold increase or decrease	Literal
Use of healthcare and nutrition services	Average hospital stay (days)	39.9	84.6	46.5	109.4	− 0.29	− 0.71	0.71 times less
	Cases referred to hospital	0.5	0.6	0.2	0.2	0.41	0.41	0.41 times more
	Curative service (primary care, treatments) utilization rate	3.9	4.7	3.3	3.7	1.15	1.15	1.15 times more
	Attendance at preschool medical consultation	5.2	7.2	3.8	5.3	0.35	0.35	0.35 times more
	Bed occupancy rate	7.8	8.0	7.2	9.5	− 0.91	− 0.09	0.09 times less
	UNTA Admissions at start of month (carry over from previous month) -Female	110.0	113.1	11.3	63.6	− 0.94	− 0.06	0.06 times less
	UNTI Admissions at start of month (carry over from previous month) -Female	7.6	3.2	1.8	2.5	− 7.12	− 7.12	7.12 times less
	Further referral to an UNTA	3.5	8.7	0.9	3.5	1.02	1.02	1.02 times more
	Moderate acute malnutrition Transferred to UNTA/ UNTI	3.2	4.5	1.1	3.8	− 0.51	− 0.49	0.49 times less
	Referred to UNTA	7.9	8.1	3.9	4.3	− 0.69	− 0.31	0.31 times less
	Referred to UNTI	3.0	6.6	1.0	3.0	0.76	0.76	0.76 times more
	Moderate acute malnutrition treated at the Supplemental Nutritional Unit	12.6	97.8	40.8	47.9	11.09	11.09	11.09 times more
Nutritional health	Attrition (failure to follow up) at the Supplemental Nutritional Unit	3.2	3.9	3.8	1.8	− 1.34	− 1.34	1.34 times less
	Discharged (healed from malnutrition)	8.6	69.8	28.0	39.1	4.53	4.53	4.53 times more
	UNTI deaths	1.3	1.7	0.6	0.5	− 15.92	− 15.92	15.92 times less
	UNTA deaths	0.7	0.6	0.3	1.0	− 1.15	− 1.15	1.15 times less
Vaccination	Vaccinated for PCV-13	7.84	9.72	6.92	8.24	0.42	0.42	0.42 times more
	Vaccinated for VAA	7.75	9.45	6.58	8.04	0.17	0.17	0.17 times more
	Vaccinated for Pentavalent 1	8.67	10.50	7.99	9.04	0.74	0.74	0.74 times more
	Vaccinated for Pentavalent 3	8.02	9.69	7.30	8.36	0.57	0.57	0.57 times more
	Vaccinated for VAR	7.65	9.38	7.20	7.99	1.20	1.20	1.2 times more
Reproductive and maternal health	Combined oral contraception pill pack	14.13	17.30	1.30	1.92	4.10	4.10	4.1 times more
	Progesterone only pill pack	6.35	3.13	3.91	0.91	0.07	0.07	0.07 times more
	Proportion of pregnant women who received the 3rd dose of folic acid	7.77	10.56	5.93	8.44	0.11	0.11	0.11 times more
	Prenatal 1 consultation rate	8.40	10.66	7.57	7.92	5.44	5.44	5.44 times more
	Prenatal 1 consultation rate at week 16	1.80	2.15	1.66	1.76	2.41	2.41	2.41 times more
	Prenatal 4 consultation rate	4.85	6.10	3.47	4.05	1.14	1.14	1.14 times more
	Tetanus vaccine coverage in pregnant women	8.69	10.38	8.54	8.66	13.24	13.24	13.24 times more
	Assisted delivery rate	6.29	7.58	4.97	5.72	0.74	0.74	0.74 times more
	Relapse with complication	0.6	0.3	0.1	0.3	− 2.25	− 2.25	2.25 times less
	Z score of -3 (weight for height) or arm circumference less than 115 mm with complications	2.7	2.8	1.5	1.7	− 0.68	− 0.32	0.32 times less
	Pregnancy related edema with complications	2.1	2.7	2.0	1.4	− 1.95	− 1.95	1.95 times less
	Pregnancy related edema	11.9	22.1	3.6	15.9	− 0.17	− 0.83	0.83 times less
	Hospital mortality rate (48 h since admission)	0.1	0.2	0.1	0.1	− 17.22	− 17.22	17.22 times less
Newborn and child health	Proportion of children aged 6 to 59 months with severe acute malnutrition	0.1	0.2		0.2	− 0.04	− 0.96	0.96 times less
	Proportion of children aged 6–23 months still breastfed	7.1	8.7	5.0	7.5	− 0.35	− 0.65	0.65 times less
	Proportion of children under 6 months being exclusively breastfed	5.5	8.2	5.0	7.7	0.01	0.01	0.01 times more
	Live births (Per 1000 inhabitants)	568.9	790.0	521.6	708.5	0.18	0.18	0.18 times more
	Proportion of children < 5 years old with acute malnutrition treated according to the national protocol: PCIMA	22.7	11.6	3.3	9.5	− 2.77	− 2.77	2.77 times less
	Proportion of children < 5 years old with diarrhea treated with oral hydrating solution, zinc	8.1	9.8	7.7	9.8	− 0.21	− 0.79	0.79 times less
	Proportion of children < 5 years with pneumonia who received antibiotics	8.2	9.9	8.1	9.8	− 0.03	− 0.97	0.97 times less
	Proportion of children < 5 years old with malaria treated according to the national protocol	7.8	9.4	8.0	9.7	− 0.09	− 0.91	0.91 times less
	Proportion of newborns put to the breast within 1 h of birth	8.1	9.7	7.6	9.5	− 0.21	− 0.79	0.79 times less
	Proportion of newborns who received 5 components of Essential Newborn Care and three examinations in the first 6 days of life	7.7	9.7	7.1	10.2	− 0.34	− 0.66	0.66 times less
	Proportion of live newborns with low birth weight	0.9	0.9	1.0	0.9	− 1.20	− 1.20	1.2 times less
	Proportion of live newborns with very-low birthweight (under 2500 g)	0.9	0.9	1.0	0.9	− 1.14	− 1.14	1.14 times less

A strong response was witnessed in the use of nutritional services: referrals and counter-referrals to UNTAs increased by 31% and 1.02-fold respectively, while referrals to UNTIs increased by 76%. Finally, there was an increase of 7.1-fold in admissions to UNTIs.

#### Positive Effects of the Program: Nutritional Health

In Kongo Central, the comparative effects seen on nutritional health from program implementation in PRO DS HZ compared to the non-PRO DS HZ included:

- The number of people cured of malnutrition increased 12.7-fold;
- Deaths in UNTAs and nutritional supplemental units were reduced by 15% and 2%, respectively;
- There was reduced attrition from nutritional supplemental units and UNTIs by 1.9-fold, and 1.6-fold, respectively.

In Ituri, the comparative effects seen on nutritional health from program implementation in the seven PRO DS HZ versus non-PRO DS HZs included:

- Deaths within UNTIs reduced by 15.9-fold;
- The number of people cured of malnutrition increased by 4.5-fold;
- Women being screened at the nutritional supplemental unit increased by 11.1-fold;
- Death rates in UNTA and nutritional supplemental unit reduced by 15% and 2%, respectively;
- Attrition from the nutritional supplemental unit reduced 1.3-fold.

#### Positive Effects of the Program: Immunization Coverage

In Kongo Central, the program improved overall immunization coverage in the three PRO DS HZ compared to non-PRO DS HZ. Increases ranged from 16 to 45% higher in immunization coverage rates (for BCG, PCV-13, VAA, Pentavalent 1, and Pentavalent 3), and a 35% decrease in the Penta 1-Penta 3 dropout rate; almost all immunization indicators were positive.

In Ituri, the program improved overall immunization coverage in the seven PRO DS HZ compared to non-PRO DS HZ. Increases ranged from 0.17-fold to 1.2-fold in vaccine coverage rates (for PCV-13, VAA, Pentavalent 1 and Pentavalent 3 and VAR).

#### Positive Effects of the Program: Reproductive and Maternal Health

In Kongo Central (Table 2), the program’s effects on reproductive and maternal health in the PRO DS HZ compared to the non-PRO DS HZ included:

- The number of pregnant women who consulted prenatal services increased 5.65-fold;
- Coverage rates increased for Prenatal Consultation 1 (1.5-fold) and Prenatal Consultation 4 (1.1-fold), as well as for assisted delivery (1.7-fold);
- Pregnancy related edema, with or without complications reduced by 62.5-fold and 30.0-fold, respectively;
- An increase of 1.63-fold in the use of oral progestin-only pills as a method of contraception.

In Ituri (Table 3), the program’s effects on reproductive and maternal health seen in the PRO DSHZs compared to the non-PRO DS HZs included:

- Hospital mortality rates (of stays over 48 h) was reduced 17.2-fold;
- The number of women who relapsed postpartum with complications was reduced 2.3-fold;
- Pregnancy related edema, with or without complications was reduced 1.9-fold and 17.0-fold, respectively.

#### Positive Effects of the Program: Newborn and Child Health

In Kongo Central, the PRO DS program improved indicators related to the health of newborns and children in the three PRO DS HZ compared to non-PRO DS HZ. The greatest impact was a 19.4-fold reduction in cases of children under the age of five suffering from acute malnutrition, followed by a 5.2-fold reduction in babies and children aged 6–59 months with moderate acute malnutrition, a result of the integrated management of acute malnutrition national program (prise en charge intégrée de la malnutrition aigue or PCIMA). Other indicators of improvement were seen in children under the age of 5 years with: diarrhea treated with oral hydrating solution (OHS) and zinc; pneumonia treated with antibiotics; malaria treated according to the national protocol, and in low-birth-weight and very low-birth-weight (under 2500 g) newborns, the proportion of babies aged 6–23 months who continued to be breastfed, and in attendance rates at preschool medical consultations (Table 2).

In Ituri, the program’s implementation improved indicators of health of newborns and children in the seven PRO DS HZs compared to non-PRO DS HZs. As in Kongo Central, the greatest impact was a 2.8-fold reduction in cases of children under the age of five suffering from acute malnutrition who were managed using PCIMA. There was a 1.2-fold reduction of low-birth-weight newborns, and a 1.1-fold reduction of very low birthweight newborns. Other indicators of improvement were seen in children under the age of 5 with diarrhea (treated with OHS and zinc), pneumonia (treated with antibiotics) and malaria (treated according to the national protocol). Improvements were seen in the number of live births, the proportion of newborns put to the breast within 1 h of birth, the proportion of babies under 6 months who were exclusively breastfeeding, the proportion of babies aged 6–23 months who continued breastfeeding, the proportion of babies and children aged 6–59 months with severe acute malnutrition, and the proportion of newborns who received five components of essential newborn care and three examinations in the first 6 days of life (Table 3).

### Unmet Outcomes of the PRO DS MEMISA Program

Despite the positive results observed with PRO DS MEMISA in both provinces, other indicators did not change. As seen in Table 4, one-third of the indicators did not show positive change. Specifically, 39.2% (29/74) and 29.2% (19/65) of the indicators were not improved through the PRO DS program in Kongo Central and Ituri provinces, respectively. In Kongo Central, 52.6% (10/19) of reproductive and maternal health indicators, 44.4% (4/9) of nutritional health indicators, and 42.9% (9/21) of newborn and child health indicators did not improve. In Ituri, all 4 indicators related to the supervision and management of HZ and health centers, and 50% (4/8) indicators related to nutritional health did not undergo any desired change.

**Table 4:** Proportion of desired results achieved through the PRO DS Memisa program

Areas	Number of indicators			Number of indicators with positive effect			Proportion of indicators with positive effect			Number of indicators without effect			Proportion of indicators without effect		
	Kongo Central	Ituri	Total	Kongo Central	Ituri	Total	Kongo Central (%)	Ituri (%)	Mean (%)	Kongo Central	Ituri	Total	Kongo Central (%)	Ituri (%)	Total (%)
Supervision and management of health zones and healthcare centers	4	4	8	3	0	3	75.0	0.0	37.5	1	4	5	25.0	100.0	62.5
Use of healthcare and nutrition services	14	13	27	9	12	21	64.3	92.3	78.3	5	1	6	35.7	7.7	21.7
Nutritional health	9	8	17	5	4	9	55.6	50.0	52.8	4	4	8	44.4	50.0	47.2
Vaccination	7	7	14	7	5	12	100.0	71.4	85.7	0	2	2	0.0	28.6	14.3
Reproductive and maternal health	19	19	38	9	13	22	47.4	68.4	57.9	10	6	16	52.6	31.6	42.1
Newborn and child health	21	14	35	12	12	24	57.1	85.7	71.4	9	2	11	42.9	14.3	28.6
Total	74	65	139	45	46	91	60.8	70.8	65.8	29	19	48	39.2	29.2	34.2

#### Implementation Difficulties That May Have Led to Unachieved Results

The analysis of documents highlighted difficulties that were encountered during program implementation in the two provinces. As well, weaknesses were identified in the PRO DS MEMISA program that may help explain unachieved results. The main findings were:

(i) the onset of the COVID-19 pandemic in March 2020. Program activities slowed down because of public health measures that had to be respected.
(ii) the growing insecurity and the internal displacement of several populations from HZ in Ituri rendered the fixed CS fees inappropriate as they were indexed to the size (number) of the population, which was in constant flux.
(iii) the withdrawal of the local bonus and occasionally the meager funding due to a large number of beneficiaries (e.g., health centers with more than 50 nurses) as well as the insignificant salary supplement due to an increase in staff being assigned in the health zones and health centers supported by PRO DS Memisa.
(iv) the precarious nature of the zonal pharmacies that were to ensure drugs stored in Kongo Central would be packaged and repackaged.
(v) there was resistance to the paradigm shifts and signing of agreements that would introduce a comprehensive approach to patient care.

### Weaknesses in the Program that Potentially Led to Unachieved Results

Based on observations of our research team in the field and from those who were collecting data, certain weaknesses in the PRO DS Memisa program were identified. These included:

(i) The application of a fixed fee in health centers without considering the movement of populations;
(ii) The same payments (fees) were made to health centers and to the referred health centers, even though the latter function as hospitals;
(iii) A lack of clarity in defining indigent persons without a study to identify the criteria for indigence;
(iv) The lack of training for healthcare personnel;
(v) Poor integration of Water, Sanitation and Hygiene (WASH) into development programs;
(vi) Weak community participation that hindered integration of the community liaison officers in the health centers;
(vii) Failure to consider family history as documented in the family medical history files;
(viii) The lack of equipment for buildings (especially in Kongo Central during the first phase of PRO DS 1).

## Discussion

The objective of this study was to evaluate the social impact of the PRO DS Memisa program that aims to strengthen healthcare systems in the DRC.

In the following paragraphs, we discuss the results obtained by PRO DS Memisa in relation to (1) the supervision and management of the HZ and CS; (2) the use of health and nutritional services; (3) nutritional health; (4) immunization; (5) reproductive and maternal health; (6) newborn and child health; and (7) program weaknesses and limitations.

1. Supervision and Management of Health Zones
  The health purchasing approach deployed by Memisa through the PRO DS program in supported HZ made it possible to measurably improve the supervision of CS by the HZ management teams in Central Kongo, the holding of ECZ and CODESA meetings. These findings corroborate those of other studies [12, 13]. Supervision of and within health centers improves the quality of services offered to the population and reaches national standards [14, 15].
  In Ituri, on the other hand, no changes were observed in CS supervision or in the overall management of the HZ. During the PRO DS implementation period, Ituri experienced increasing insecurity, which sometimes made it difficult for the HZ management team to travel to health areas. The PRO DS program improved CODESA performance, and ultimately the community participation which is recognized in population health as an essential driver of sustainable improvement [16].
2. Health Services Utilization
  At the health service level, the purchase of services improved rates of postoperative infections and hospital referrals, the average hospital length of stay, rates of bed occupancy, curative service use (primary care and treatments), attendance at prenatal consultations, and hospital mortality in those admitted more than 48 h prior. These results have been obtained in similar programs [14, 17, 18]. In Ituri, as in Central Kongo, the purchase of services raises the issue of equity in access to healthcare [19]. First, services are limited to 10 out of the 67 HZs (3/31 in Central Kongo and 7/36 in Ituri), and, secondly, not all components of healthcare can be acquired using a health service purchasing approach.
3. Nutritional Health and Use of Nutritional Services
  The program's positive effect on the recovery of malnourished individuals, reduction in deaths in UNTAs and UNTIs, and reduced attrition from UNTAs and UNTIs corroborate the findings of other programs [20–23]. Nimpagaritse et al. took into consideration indicators at the community level (food access and cooking), the health center level (malnutrition screening and nutrition education), and the hospital level (medical management, complication management, hospitalization for malnutrition) [24] thus providing a holistic approach to malnutrition management.
4. Immunization
  Vaccination indicators improved because of the program, in particular the increased rates of vaccination coverage for BCG, PCV-13, VAA Pentavalent 1 and Pentavalent 3 and VAR and reduced rates of booster shot attrition for Penta 1-Penta 3. Vaccination is an important component supported by the program, being recognized as one of the pillars of infant mortality prevention. These results corroborate those obtained in similar studies [20, 25, 26] including in Nigeria where performance-based financing has contributed to polio eradication [27]. However, in some studies performance-based financing has not resulted in improved immunization coverage [28, 29].
5. Reproductive and Maternal Health
  The positive effects of the program are largely reflected in reproductive and maternal health outcomes. In Kongo Central, there was a substantial increase in prenatal consultation and reduction in pregnancy related edema (with or without complications). In Ituri, the program resulted in reduced hospital mortality, and lower rates of pregnancy related complications or relapse of edema. Effects were not observed for the number of pregnant women who: consulted prenatal services, received a third dose of folic acid or tetanus vaccines, or who consulted for prenatal consultations #1 and #4. The coverage rate for assisted delivery was also not significantly affected by the program.
6. Newborn and Child Health
  In Kongo Central, the program brought about a reduction in the number of acutely malnourished children under the age of five, and a reduction in the number of moderately malnourished children aged 6–59 months. In Ituri, fewer low-birth-weight babies were born, and there was a reduction in the number of acutely malnourished children under the age of five. Other indicators of newborn and child health were not significantly affected by the program. PRO DS has supported PCIMA, which promotes community involvement and leadership in malnutrition prevention and management strategies. This approach has proven to be effective and corroborates the results of other studies [22].
7. Barriers Encountered During PRO DS Program Implementation
  A series of barriers and limitations were identified for program implementation and success. The first of these was the appearance of the COVID-19 pandemic, which slowed program activities and adversely affected the achievement of certain indicators, including those related to maternal and child health [30, 31]. The growing insecurity and internal displacement of populations may have affected the ability of the program to provide packages that would be appropriate for the population accessing each health center. Indeed, several internally displaced groups in Ituri HZ made the packages inadequate or obsolete. Constraints also included the uncertain ability of zonal pharmacies to provide the packaging and repackaging of drugs stored in Kongo Central which may have hindered the quality of care and limited access to healthcare services [32, 33]. There was also resistance to this paradigm shift. This may have been due the strength of the stakeholders' culture, ingrained attitudes, established organizational approaches and change management models [34, 35]. Resistance to change can be mitigated by analyzing ahead of time the concerns important stakeholders may have, and by inviting key players to co-design the program prior to implementation [36]. Indeed, stakeholders should be fully involved in important decisions made during program development and implementation [37].

### Study Limitations

This study has several limitations. This work is an analysis of secondary data, which unlike primary data analysis, may have more inherent bias [38–40]. To mitigate this, many indicators (74 in Kongo Central and 65 in Ituri) were considered to have a broad spectrum of potential effects over time. Secondly, the potential impacts estimated using the double-difference method were not adjusted (via statistical or econometric modeling) in relation to variables specific to any health zone characteristics such as size, accessibility, income of households in the area, access to drinking water and electricity, the presence of programs from other technical and financial partners, or other factors. However, it was possible to obtain robust data by analyzing variation over time by comparing the baseline (base year) and the periods since the project began. Finally, health zones did not have comparators matched between the PRO and non-PRO DS Memisa according to their context, demographics, or geographic location. However, in both provinces studied, the PRO DS Memisa HZ were compared over time to all the non-PRO DS Memisa HZ.

## Summary and Recommendations

This study demonstrated measurable positive health impacts from the PRO DS Memisa comprehensive and integrative health system strengthening program in the DRC where living conditions remain far below world standards. Further work should evaluate the medium- and long-term effects of this and similar programs to be able to gauge sustainability, scalability and future investment requirements. Qualitative studies implemented alongside quantitative analyses may provide a deeper understanding of the barriers that exist to the implementation and uptake of health-based initiatives.

In the absence of any existing policy for the sustainability for the PRO DS Memisa program at the end of its mandate, the following ten elements deserve to be capitalized on to ensure comprehensive, high-quality healthcare for the population through the provision of services and by strengthening nutrition: (1) Maintain the comprehensive care approach through the purchase of services, the third party payer, and flat-rate pricing (confirm the flat-rate pricing beforehand); (2) Maintain a high-quality approach acquired by the HZ and CS and maintain intensive supervision of the DPS; (3) Maintain the banking system and rigor in the administrative and financial management in the health centers and health zones; (4) Continue to develop the management, operational action and drug management plans; (5) Encourage community dynamics to strengthen the supply and promote mutual aid to continue the same strategy; (6) Preserve what has been achieved by maintaining equipment, built facilities, rolling stock, etc.; (7) Maintain the internal and external hygiene of the hospital and healthcare centers through a high-quality approach; (8) Keep the principle of distribution key with progressive adjustments of the proportions in four categories: medication, operations, additional remuneration, and provisions; (9) Encourage a principle of cooperation between the HZ in the management of medication supply at the regional distribution centers that serve the provinces; and (10) Raise awareness for, interact with and educate the population through community.

**Table:** Abbreviations

Original French term	English term equivalent
VAA: le vaccin antiamaril	Yellow fever vaccine
BCG: vaccin de calmette et guérin	BCG: Tuberculosis vaccine
CAAMEKI: centrale d’achat et d’approvisionnement de médicaments essentiels de Kisantu	CAAMEKI: central purchasing and supply of essential drugs in Kisantu
CADIMEBU: central d’approvisionnement et de distribution de médicament essentiels de Bunia	CADIMEBU: central supply and distribution of essential drugs in Bunia
Centrale de Distribution Régionale	Regional distribution center
CODESA: comité de développement de l’aire de santé	CODESA: health area development committee
CPN: consultation prénatale	Prenatal consultation
CPS: consultation préscolaire	Preschool consultation
CS: Centre de santé	Health centre
DPS: Division provinciale de la santé	Provincial health division
ECZ: équipe cadre de la zone de santé	Health zone management team
EUP-FASS: établissement d’utilité publique-fond d’achat des services de sante	EUP-FASS PO: public utility establishment-fund for the purchase of health services
HGR: hôpital générale de référence	HGR: general referral hospital
MUSAKIS-FASS: mutuelle de santé de Kisantu- fond d’achat des services de santé	MUSAKIS-FASS: Kisantu mutual health insurance fund-health services procurement fund services
PCIMA: prise en charge intégrée de la malnutrition aigue	PCIMA: integrated management of acute malnutrition (national program)
PCV-1-3: vaccin contre le pneumocoque-13	PCV: pneumococcal vaccine-13
PRO DS: programme de renforcement de l’offre et de développement de l’accès aux soins de santé	PRO DS: program to strengthen supply and develop access to health care
UNTA: unité nutritionnelle thérapeutique ambulatoire	Outpatient Therapeutic Nutritional Unit
UNTI: unité nutritionnelle thérapeutique intensive	Intensive Therapeutic Nutritional Unit
VAR: Vaccin contre la varicelle	VAR: Varicelle Vaccine
ZS: zone de santé	HZ: health zone

## Data Availability

The datasets created and/or analysed during the current study are available from the corresponding author on reasonable request.
